# Insights from the Complete Genome Sequence of Bacillus circulans GN03, a Plant Growth-Promoting Bacterium Isolated from Pak Choi Cabbage (Brassica chinensis L.) Root Surface

**DOI:** 10.1128/MRA.00581-20

**Published:** 2020-08-20

**Authors:** Lijun Qin, Xingyong Yang, Hong Shen

**Affiliations:** a Biological Science Research Center, Southwest University, Chongqing, China; b College of Life Science, Chongqing Normal University, Chongqing, China; c College of Resources and Environment, Southwest University, Chongqing, China; University of California, Riverside

## Abstract

A plant growth-promoting rhizobacterium, Bacillus circulans GN03, was isolated from the root surface of pak choi cabbage. Here, we report the whole-genome sequence of the GN03 strain, which includes a circular chromosome (5,217,129 bp; GC content, 35.64%) and a plasmid (181,705 bp; GC content, 31.62%).

## ANNOUNCEMENT

Plants are constantly exposed to a great variety of microorganisms inhabiting the rhizosphere and the epiphytic and endophytic phyllosphere at every stage of their life cycle (1). They may also establish mechanisms to resist, tolerate, or utilize the microorganisms under stimulation or induction by a variety of factors, including plant growth-promoting rhizobacteria (PGPR) (2). PGPR with the ability to colonize plant root surfaces or the rhizosphere can directly or indirectly promote or regulate plant growth. At present, more than 20 species and genera of PGPR strains have been identified, including *Agrobacterium*, *Azotobacter*, and *Bacillus*. (3, 4). Of these bacteria, *Bacillus* is one of the largest bacterial genera worldwide (5) and can be widely isolated from crops, vegetables, and so on, either as rhizobacteria or as endophytes (1).

Bacillus circulans is a Gram-positive, rod-shaped, aerobic or anaerobic bacterium with rough colonies on agar-solidified culture medium (6) and was first isolated and described in 1890 by Jordon (7). *B. circulans* GN03 was isolated from the root surface of pak choi cabbage (Brassica chinensis L.) at Beibei District (30°26′12″N, 106°26′25″E), Chongqing, China (8). This strain was demonstrated to promote the growth of various plants (8). To further elucidate its mechanism of plant growth promotion, we describe the complete genome sequence of the bacterium in this study.

The isolated GN03 strain was cultured in nutrient broth by shaking overnight at 120 rpm and 28°C and was identified as *B. circulans* by colony PCR for 16S rRNA gene amplification using the bacterial universal primers (27F/1492R). A single colony of strain GN03 was grown in 10 ml of Luria-Bertani broth at 37°C overnight (20 h) with shaking (120 rpm). The genomic DNA from enriched bacterial cells was extracted using the cetyltrimethylammonium bromide (CTAB) method (9) and sequenced using the PacBio RS II system (Guangzhou Gene Denovo Biotechnology Co., China). The mean read length was 9,173 bp, the *N*_50_ read was 12,700 bp, and the genome coverage was 169×. The whole-genome sequence was assembled using HGAP 3.0 (10) and trimmed by quality with filtering steps that removed reads with a read length less than 100 and removed reads with an average read quality value less than 0.80. An assembly contig of 5,217,129 bp was finalized as the circular chromosome and a contig of 181,705 bp as the plasmid. The mean GC content of the circular chromosome was 35.64%, which was similar to the average across *B. circulans* genomes (length, 5.09 Mbp; mean GC content, 35.5%) recorded by the NCBI, and the mean GC content of the plasmid was 31.62%.

Using rRNAmmer (version 1.2; parameters, -S bac -m tsu, lsu, ssu) (11) to predict rRNAs, 11 copies of 5S rRNAs, 16S rRNAs, and 23S rRNAs were predicted. Using tRNAscan (version 1.3.1; parameters, ‘-S bac -m tsu, lsu, ssu) (12), 84 copies of tRNAs were predicted in the genome. We also predicted 25 transposons in the chromosome and 3 transposons in the plasmid using TransponsonPSI (http://transposonpsi.sourceforge.net) (version 20100822; parameters, -e 1e-5); furthermore, 23 gene islands were predicted in the chromosome and 1 gene island in the plasmid using IslandViewer (version 4) online (http://www.pathogenomics.sfu.ca/islandviewer/). We also searched for CRISPRs using CRISPRfinder (version 4.2.17; parameters, -mismDRs 20 -minDR 23 -maxDR 55) online (http://crispr.i2bc.paris-saclay.fr/Server/), but we did not find any CRISPR sequences. The ORFs were predicted using GeneMarkS (13). Default parameters were used for all software except where noted. A general description of the genome is shown in Table 1.

**TABLE 1:** General information on the *B. circulans* GN03 genome

Attribute	Data for:	
	Chromosome	Plasmid
Genome size (bp)	5,217,128	181,705
GC content (%)	35.64	31.62
No. of protein-coding genes	5,324	193
No. of rRNAs	11	0
No. of tRNAs	84	0
No. of transposons	25	3
No. of gene islands	23	1
NCBI accession no.	CP053315	CP053316

### Data availability.

The project data for Bacillus circulans strain GN03 have been submitted under GenBank accession number CP053315.1 and BioProject accession number PRJNA630659. The raw data have been deposited in the SRA under accession number SRR11831569.
